# Acute systemic embolism due to an idiopathic floating thrombus of the thoracic aorta: success of medical management: a case report

**DOI:** 10.1186/s13104-015-1149-1

**Published:** 2015-05-02

**Authors:** Yves Ghislain Abissegue, Youssef Lyazidi, Hassan Chtata, Tarik Bakkali, Mustapha Taberkant

**Affiliations:** Department of Vascular Surgery, Mohammed V Military Hospital, Mohammed V University, Dr Abissegue Yves S/C ERSSM BP 1044 Rabat Océan Maroc, Rabat, Morocco

**Keywords:** Acute systemic embolism, Floating thrombus, Thoracic aorta, Medical management

## Abstract

**Background:**

Idiopathic thoracic aortic mural thrombi are rare. They can be responsible for dramatic systemic embolization. Early treatment is imperative because of their high morbidity and mortality rate.

**Case presentation:**

A 55-year-old previously healthy Moroccan male came in an array of acute right lower limbs pain and abdominal sensibility. Severe systemic embolism involving the lower extremities, spleen, kidney, and digestive tract, due to an idiopathic mural thrombus of the thoracic aorta was diagnosed. He received medical treatment leading to the complete disappearance of the thrombus and the effects caused by the latter.

**Conclusions:**

When faced unexplained peripheral embolization, research for a thrombus of the thoracic aorta should be performed. Medical treatment should be considered for its management, especially in patients with high surgical risk.

## Background

Thoracic Aortic mural thrombus (TAMT) accounts for 0.9% of all aetiologies of peripheral arterial embolism and is responsible for significant morbidity and mortality [1]. Its most frequent origins are intra cardiac or intra aneurismal thrombus, atherosclerotic aortic lesions, venous paradoxical embolization, malignant diseases, trauma, coagulation disorders, and certain systemic and rheumatic diseases.

Idiopathic or isolated thoracic aortic mural thrombus (IAMT) developing in the absence of aortic lesion, cardiac, hematologic or haemostatic disorders, are much more rare [2]. Currently, the common use of computed tomography (CT), transesophageal echocardiography (TEE) and magnetic resonance imaging (MRI) has facilitated the early diagnosis of this condition, which is still without a set treatment [1,3]. We recount the medical management of a patient with an IAMT revealed by severe peripheral and visceral embolism. We will then recall the diagnostic and therapeutic methods recommended in the treatment of this rare condition.

## Case presentation

A 55-year-old Moroccan male came into the emergency department with abdominal pain, vomiting, and lower right limb pain. He had consulted two days earlier at a different health centre and was prescribed an anti-inflammatory and anti spasmodic. He reported no improvements. He was a former smoker, weaned for 10 years, and had a history of hypothyroidism treated with Levothyroxine.

Physical examination on admission found coldness and paresis of the right ankle and foot, associated with the absence of popliteal, posterior and pedal tibia pulses. The left leg showed no anomaly. His abdomen was sensitive but without signs of peritonitis. Blood pressure was 138/85 mmHg with sinus tachycardia at 134 beats/min.

Arterial and venous Duplex ultrasonography of the lower limb, showed thrombosis of the popliteal arteries and tibial tripod. There were no signs of deep venous thrombosis. Transthoracic echocardiography (TTE) showed neither valvular nor intra cavitary anomalies. Ventricular ejection fraction was preserved. Laboratory tests noted elevated muscle enzymes; ASAT at 297 IU/L, CPK 11902 IU/L, LDH at 623 IU/L, (troponin I was normal), white blood cells (WBC) count at 13×10^9^/L and high C-reactive protein (CRP) level at 500 mg/L. Renal function was impaired; urea 13.6 mmol/l, creatinine 221 μmol/l, measured creatinine clearance at 27 ml/min. His diuresis was conserved. Coagulation and crasis tests were normal. Blood samples were taken for assessment of thrombophilia (protein S, protein C, Antithrombin III).

Embolectomy of the sub articular popliteal artery was performed under local anaesthesia. Fresh thrombus was extracted from the popliteal artery with recovery of good flows. But embolectomy of the leg arteries revealed an old and adhering thrombus with poor reflux. Fasciotomy of the leg completed the surgery. He was subsequently admitted in intensive care (ICU) and received sodium heparin (500 IU/Kg/24 h) using an automatic syringe, antibiotics, an analgesic and a proton pump inhibitor (PPIs). Oral feeding was stopped.

**Figure 1:**
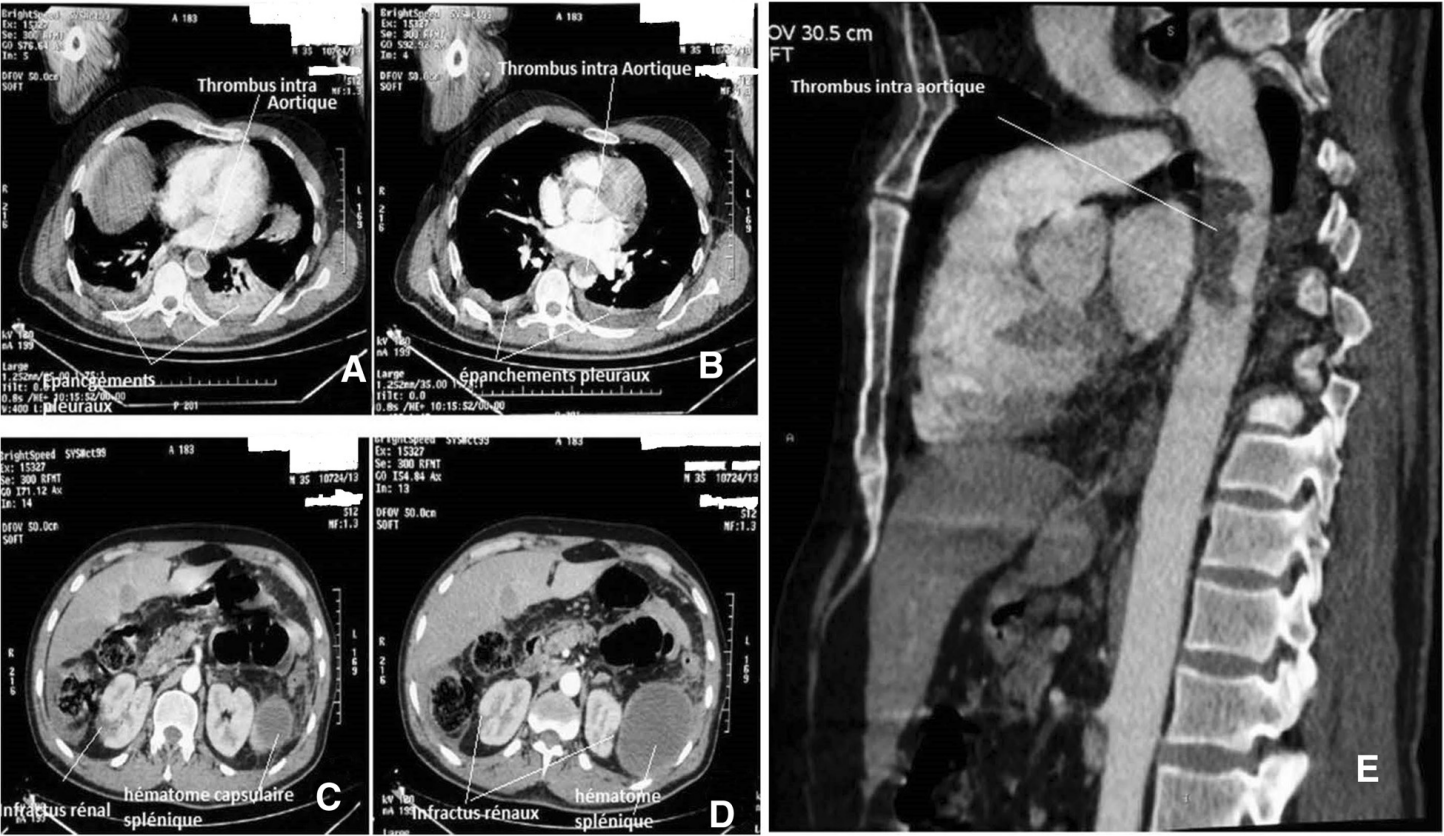
CT angiography showing the thoracic aorta of normal caliber without parietal atherosclerotic lesion, with an intra-aortic thrombus. Axial section; **A**, **B**. Sagittal reconstruction; **E**. Kidney infarct and peri-splenic hematoma, axial section **C**, **D**.

**Figure 2:**
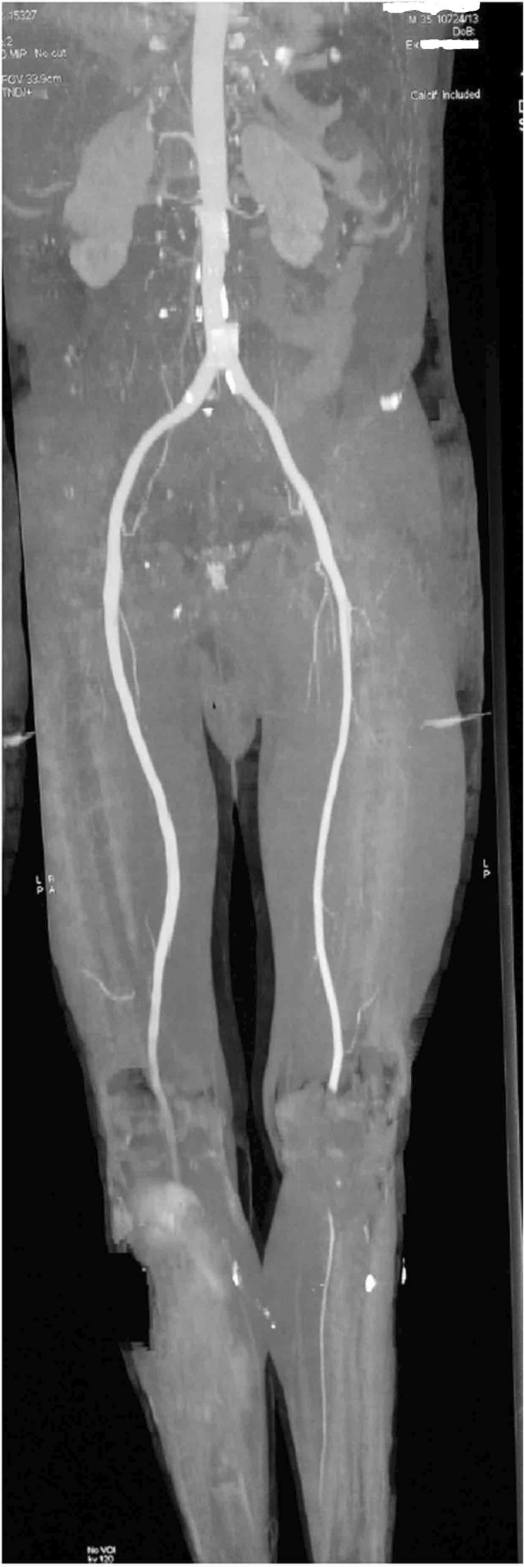
CT angiography of the lower limbs showing. Right; poor patency of the operated arterial axis, and Left; a change in contrast at the beginning of the popliteal artery.

The next morning, coldness of the left lower limb was found with the abolition of the distals pulses. We realized a thoraco-abdominal and lower limb angio CT. It showed the thoracic aorta of normal size with no parietal atherosclerotic lesion and containing a thrombus measuring 43 mm whose upper pole was located at 15 mm of the aortic isthmus (Figure 1A, B, E), and signs of renal and splenic infarction, very thin colonic wall not enhanced by the contrast agen, suggestive of ischemia (Figure 1B, C). The arteries of the lower limbs were occluded beginning at the popliteal artery (Figure 2).

Surgical treatment of the aortic thrombus was proposed, but patient and his family who were made aware of the surgical risks refused. The dose of heparin was increased (700 IU/Kg/24 h), associated with Naftidrofuryl (Praxilene® 200 mg) 2 tablets/8 h. On D_4_, amputation of the right thigh was carried out because of the extension of the lesions on this limb, a constant hyperleukocytosis and a high CRP level. However the coldness of the lower left limb had disappeared.

**Figure 3:**
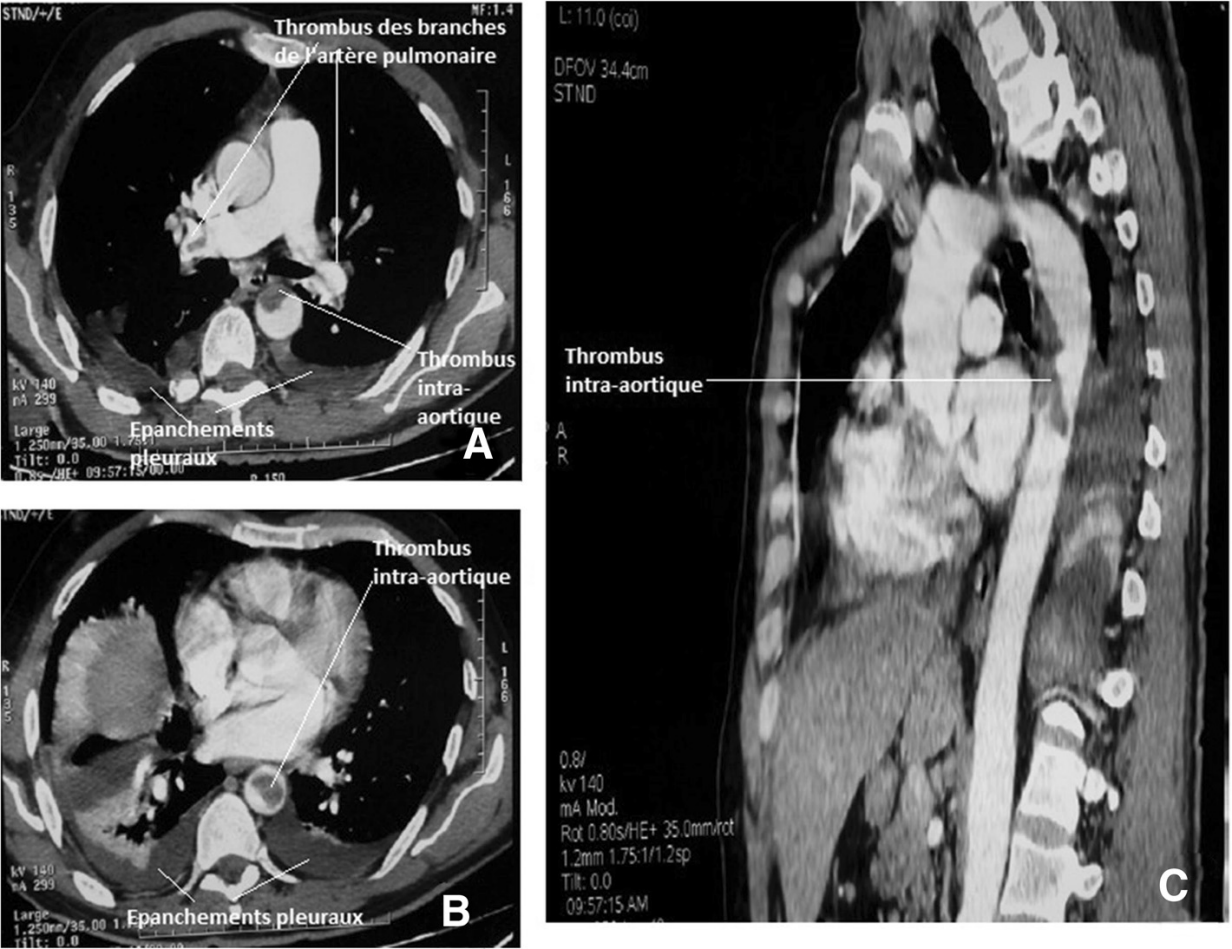
CT angiography showing a thrombus in the pulmonary artery branches. Axial section **A**; bilateral pleural effusions, Axial **A** and **B**; persistence of intra aortic thrombus. Axial section **A** and **B** and sagittal reconstruction **C**.

From D_10_ we finally began the introduction of oral anticoagulation (OAC) by vitamin K antagonists, Acenocoumarol (sintrom® 4 mg). INR target (2–3) was obtained after 3 days of overlap. We didn’t introduce them earlier because the surgical option was not abandoned and the decision for medical treatment had not yet been taken. Renal function was stabilized, as well as WBC count and CRP levels. TEE could not be realized due to technical reasons, so we did another CT scan (Figure 3), which showed the persistence of intra aortic thrombus, pleural effusions, and the appearance of thrombus tracks in the right and left pulmonary artery branches.

At D_21,_ his condition improved and he was transferred to the inpatient ward. One week later, at his request, he was discharged but kept under treatment, which comprised of OAC, antiplatelet and statin for hypercholesterolemia discovered during his hospitalization.

A new thrombophilia assessment was carried on consultation at the department of Internal Medicine. No abnormalities were found.

**Figure 4:**
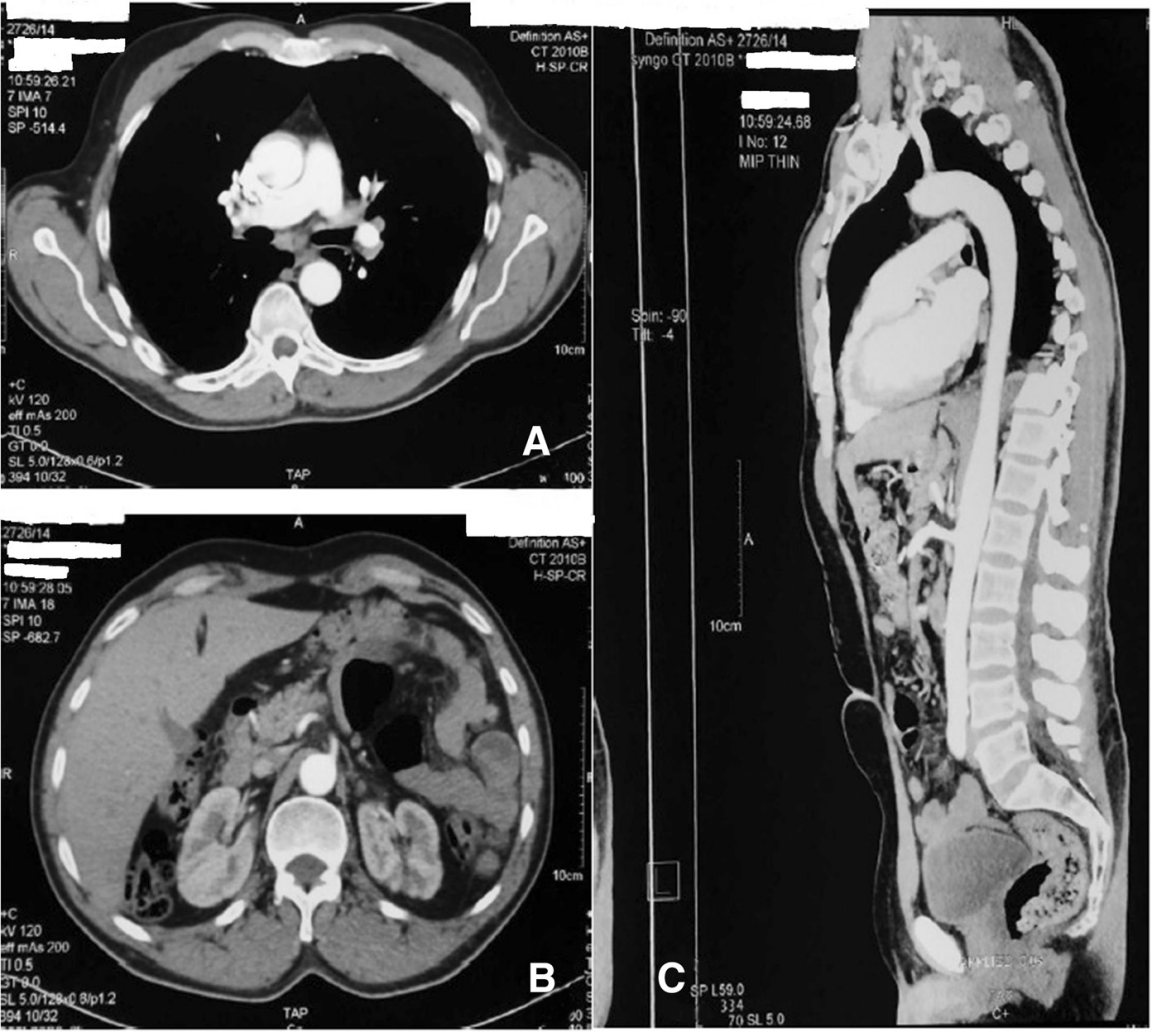
CT angiography showing a complete reversal of the thoracic aorta with disappearance of thrombus. Axial section **A** and sagittal reconstruction **C**. Notch sequelae of kidney infarct, axial section **B**.

CT scan realized 2 months later showed a complete recanalization of the thoracic aorta with disappearance of the thrombus (Figure 4). 10 months after this episode, the patient presented no embolic recurrence. His thigh was fitted and he is still under OAC.

## Discussion

The usual mode of revelation of IAMT is critical limb ischemia often associated with abdominal pain in patients without obvious risk factors for thromboembolic diseases [1,2]. Embolic source must be quickly identified and localized to avoid a potential recurrence or fatal evolution.

TEE is considered the examination of choice for the diagnosis and evaluation of IAMT. Its diagnostic efficiency would be 50% in patients with peripheral arterial embolism in the absence of significant cardiac disease [3]. It would also provide information on the size of the mass, its morphological layout (pediculate or sessile), and its dynamic or static characteristics, location, and appearance of the aortic wall. This has a great importance for the therapeutic choice. Its main limitations are incomplete visualization of the aorta and the need for it to be done under general anaesthesia in unstable patients [4].

Angio CT and MRI are also very effective for the diagnosis of aortic masses. These tests allow a study of the entire aorta and to eliminate an aortic aneurysm or atherosclerotic or calcified lesions. MRI may be more sensitive in detecting a thrombus.

Presently, it is recommended to use angio CT or MRI combined with TEE for the detection and evaluation of thrombus of the thoracic aorta [1,4]. In addition to the morphological examinations, haematological evaluation and cardiovascular assessment should be performed [5]. In our case, the etiological investigation found no hematological abnormalities. His only cardiovascular risk factors were his former smoking, and hypercholesterolemia uncovered during hospitalization.

There is still no consensus on the treatment of IAMT. According to Fayad et al. [6], aortic surgery should be considered as first-line treatment, especially for patients with a high risk of relapse. Treating with anticoagulation only would be associated with a higher recurrence risk and incidence of complications such as loss of a limb.

The techniques used are varied and often complex ranging from thrombectomy/embolectomy by sternotomy or left thoracotomy, often with the establishment of an atrio-femoral shunt or cardiopulmonary bypass [7]. Endovascular procedures that allow the exclusion of thrombus by stent-graft are under development [8,9]. They require, however, a careful selection of patients in relation to the location of thrombus and the anatomical conditions of the aorta. Their main complication is the increased risk of thrombus migration when navigating in the aorta or during stent-graft deployment. These options represent sensitive procedures, difficult to implement in units not trained in this type of interventions.

The choosing of medical treatment as a first resort is making a comeback. Whether anticoagulation or systemic thrombolysis [5,10,11], these options would help to reduce morbidity and the length of stay at the hospital in patients for whom they are suitable [5].

In the study of Turley [5], 83% patients treated with anticoagulation only and the single patient treated by thrombolysis, experienced a complete resolution of the thrombus with a single case of complication being a major amputation.

Others suggest treatment with heparin when finding a mobile thrombus in the aorta, and reassessment by TEE after 15 days of treatment [12]. Surgical treatment will be offered only after failure of medical treatment to patients in whom other causes of embolic events have been excluded. Selection criteria include young age, recurrent embolic events, and the high mobility of the thrombus. After thrombectomy, long-term treatment by anticoagulants and regular monitoring by TEE or angio CT, an MRI is necessary due to the high risk of relapse.

In our case, due to the highly volatile state of the patient, as well as surgical risks, the medical option seemed to have been the best choice in light of the results obtained.

## Conclusions

IAMT are rare conditions that can be associated with embolic events in a patient with no predisposing factors. Despite the development of surgical and endovascular procedures, medical care should be considered in high surgical risk patients.

## Consent

Written informed consent was obtained from the patient for publication of this case report. Copies of the written consent are available for review by the Editor-in-Chief of this journal.

## Abbreviations

TAMT: Thoracic aortic mural thrombus
IAMT: Idiopathic (or Isolated) thoracic aortic mural thrombus
TEE: Transesophageal echocardiography
CT: Computed tomography
MRI: Magnetic resonance imaging
ECG: Electrocardiogram
TTE: Transthoracic echocardiography
WBC: White blood cells
CRP: C-reactive protein
ASAT: Aspartate aminotransferase
CPK: Creatine phosphokinase
LDH: Lactate dehydrogenase
ICU: Intensive care unit
PPIs: Proton pump inhibitors
OAC: Oral anticoagulation
INR: International normalized ratio
